# Identification of Differentially Expressed Hub Genes Associated With Immune Cell Recruitment in Claudin-Low Breast Cancer

**DOI:** 10.3389/fonc.2022.848206

**Published:** 2022-03-11

**Authors:** Yange Wang, He Shi, Yulu Zhang, Qian Zeng, Tingmei Chen, Chengsen Chai

**Affiliations:** Key Laboratory of Clinical Laboratory Diagnostics (Ministry of Education), College of Laboratory Medicine, Chongqing Medical University, Chongqing, China

**Keywords:** Claudin-low breast cancer, differentially expressed genes, cytokine, chemokine, tumor-infiltrating immune cell

## Abstract

Breast cancer (BCa) is the most common malignancy in women and claudin-low breast cancer (CL-BCa) is a newly identified BCa subtype characterized by low expression of claudin 3&4&7. However, the hub genes associated with the recruitment of immune cells into CL-BCa were rarely described. This study aimed at exploring the differentially expressed hub genes associated with tumor-infiltrating immune cells in CL-BCa by a multi-approach bioinformatics analysis. The top 200 genes associated with CL-BCa were screened in the METABRIC dataset; the PPI network was constructed using STRING and Cytoscape; tumor-infiltrating immune cells were analyzed by TIMER 2.0; and the correlation of feature cytokines and claudins on survival was examined in METABRIC and TCGA datasets. Consequently, we found that the fraction of tumor-infiltrating immune cells, especially CD8+T cells and macrophages, increased in the CL-BCa. Differentially expressed cytokines (CCL5, CCL19, CXCL9 and CXCL10) were related to the overall survival, and their expression levels were also examined both in tumor tissues of CL-BCa patients by IHC and in typical CL-BCa cell lines by qPCR. Moreover, the BCa patients with low expression of these differentially expressed claudins (CLDN8, CLDN11 and CLDN19) showed a worse overall survival. This study sheds light on molecular features of CL-BCa on immune microenvironments and contributes to identification of prognosis biomarkers for the CL-BCa patients.

## Introduction

Breast cancer (BCa) is the most common malignancy worldwide in women with 2 million diagnosed cases and over 627,000 death cases in 2018 (1). Initially, breast cancer was classified into different subtypes based on hierarchical clustering of gene expression (2). Owing to the limitations of hierarchical clustering for the classification of individual samples, prediction analysis of microarray 50 (PAM50) was developed to identify intrinsic subtypes. Among these subtypes, four subtypes (namely, luminal A, luminal B, HER2+ and basal) exhibited unique patterns of gene expression (3). However, adequate tissue samples for microarray gene expression profiling (GEP) were required for these taxonomies (4, 5). This limitation led to the development of immunohistochemical (IHC) surrogate definitions for identifying the molecular subtypes of breast cancer (6). Unfortunately, neither GEP-based nor IHC-based taxonomy was highly accurate for clinical diagnosis and some BCa patients could not be categorized into molecular subtypes based on these taxonomies. Therefore, it was necessary to portray BCa subtypes for enhancing the accuracy of clinical diagnosis by identifying their potential gene markers using bioinformatics approaches.

Recently, novel subtypes were identified by studies with a larger sample volume of datasets. The claudin-low breast cancer (CL-BCa) was identified as one of novel BCa subtypes, and its hallmark was the low expression level of critical cell adhesion molecules, including claudin 3&4&7, Occludin and E-cadherin (7). Since tumor cells of CL-BCa exhibit some mesenchymal traits and stem cell features, CL-BCa is considered as the most primitive breast tumor (8). Kay Dias et al. studied panels of antibodies to facilitate the identification of claudin-low tumors and then identified panels of genes associated with phenotypes of claudin-low subtype (9). However, specific genes in CL-BCa associated with the characteristics of immune cell infiltration were still not clarified clearly.

In this study, the differentially expressed genes and their related pathways in CL-BCa relative to other BCa subtypes were analyzed by using multiple datasets and then hub genes related with tumor-infiltrating immune cells were identified; then, we analyzed the association between these DEGs with overall survival and eventually examined the expression of featured DEGs in CL-BCa cells by qPCR and CL-BCa tissues by IHC. In summary, our finding revealed differentially expressed hub genes associated with immune cell recruitment in CL-BCa and might contribute to identification of prognosis biomarkers for CL-BCa patients.

## Materials and Methods

### Data Acquisition

The expression profiles and clinical information of BCa patients were downloaded from TCGA *via* GDC Legacy Archive (113 normal cases and 1098 BCa cases with FPKM data). The expression profiles and clinical information of the METABRIC BCa cohort were downloaded from cBioPortal (https://www.cbioportal.org/). The gene set of cytokine activity was obtained from GSEA (https://www.gsea-msigdb.org/gsea/index.jsp). The GEPIA (http://gepia.cancer-pku.cn/) website was used to detect gene expression levels in TCGA.

### Hub Gene Selection and Protein–Protein Interaction Network Construction

A gene co-expressing analysis was performed by screening mRNA profiles in the METABRIC dataset using the WGCNA R package (10). BCa cases in the METABRIC cohort were classified into six groups based on clinical information (claudin-low: 199 patients; basal: 199 patients; HER2+: 220 patients; Luminal A: 679 patients; Luminal B: 461 patients). The top 200 genes in the most significant module were extracted to perform protein–protein interaction network (PPI) construction *via* the STRING online tool (Search Tool for the Retrieval of Interacting Genes, https://string-db.org/, version: 11.0). The PPI network was input to Cytoscape software (11). The most significant module of the PPI network was detected by the MCODE which was constructed for clustering a network based on topology to determine intensively connected Regions (12), and then hub genes in this module were found by using CytoHubba (13).

### Differentially Expressed Gene Screening and Gene Set Enrichment Analysis

Differentially expressed genes (DEGs) were analyzed in four-pair-wise comparisons by the R package of linear models for microarray data (“limma”) (14). Pathway enrichment analysis was conducted on the DEGs with |log2-fold change (FC)| ≥ 1 using Kyoto Encyclopedia of Genes and Genome (KEGG) *via* the “clusterProfiler” R package. DEGs with log2-fold change (FC) ≥ 1.5 intersected with the gene sets associated with cytokines from Molecular Signatures Database (MSigDB). The TCGA-BRCA dataset was divided into two groups by the median of the expression levels of CLDN3/4/7, OCLN and CDH1 (172 cases in claudin-low TP and non-claudin-low TP, respectively). Claudin-low BCa were identified in the Oslo2 and TCGA-BRCA databases by the nine-cell line claudin-low predictor using the R package of “Genefu” (15), and then expression levels of interested cytokines and claudin proteins were also confirmed in CL-BCa of the Oslo2 dataset and TCGA-BRCA database (Oslo2 dataset: 13 CL-BCa cases and 352 non-CL-BCa cases; TCGA-BRCA: 33 CL-BCa cases and 1058 non-CL-BCa cases).

### Tumor-Infiltrating Immune Cell Analysis and Outcome Analysis in Kaplan–Meier

Infiltration of immune cells in 1,758 BCa patients of the METABRIC dataset were investigated using the R package of “immunedeconv” (16) and web server of TIMER 2.0 (http://timer.cistrome.org/) which is an online tool for assessing the specific gene(s) associated with tumor immune infiltrating cells (17). Relative proportions of tumor-infiltrating immune cells were extracted and classified according to the different algorithms. Combined with expression levels in the TCGA dataset, all possible target genes were selected for survival analysis. Survival analysis was conducted by the “survminer” and “survival” R package. Kaplan–Meier survival curves were conducted for each group, respectively, and compared statistically using the log-rank test for testing the null hypothesis of no difference in survival between two or more independent groups. Multivariable Cox regression analyses were conducted by the “forestplot” R package. Pearson correlations were calculated with the “cor.test” package in R, and correlation plots were prepared with the “ggpubr” and “corrplot” packages in R. The Wilcoxon test and Kruskal–Wallis H test were used to determine significance for non-parametric data; p value <0.05 was considered statistically significant. All analyses were conducted using R version 4.0.2.

### Quantitative Polymerase Chain Reaction

The relative expression of DEGs was examined by quantitative polymerase chain qPCR (Bio-Rad CFX Connect™ Optics Module) and was normalized to the expression of β-actin using the 2^-ΔΔCt^ method according to a previous study (18). Total RNA was extracted with TRIzol reagent (TaKaRa, Japan, Cat#9109), and cDNA was reverse transcribed subsequently with the PrimeScript RT Reagent Kit (TaKaRa, Japan, Cat#RR037A). qPCR was then performed using a SYBR Premix Ex Taq II (TaKaRa, Japan, Cat#RR820A) according to the manufacturer’s instructions. The sequences of qPCR primers used were presented as follows: CCL5, 5′-CAGCAGTCGTCCACAGGTCAAG-3′ (forward) and 5′-TTTCTTCTCTGGGTTGGCACACAC-3′ (reverse); CCL19, 5′-AGCCTGCTGGTTCTCTGGACTTC-3′ (forward) and 5′-AGGGATGGGTTTCTGGGTCACAG-3′ (reverse); CXCL9, 5′-TCTTGCTGGTTCTGATTGGAGTGC-3′ (forward) and 5′-GTCCCTTGGTTGGTGCTGATGC-3′ (reverse); CXCL10, 5′-AACTGTACGCTGTACCTGCATCAG-3′ (forward) and 5′-ACGTGGACAAAATTGGCTTGCAG-3′ (reverse).

### Immunohistochemistry

The expression of DEGs (CCL5 and CCL19) in BCa patients was examined by immunohistochemistry (IHC). Antibodies used for IHC were CCL5 (Cat#abs136939, Absin, Shanghai, China), CCL19 (Cat#abs149041, Absin), iNOS (Cat#GB11119, Servicebio, Wuhan, China), and CD8 (Cat#GB13068, Servicebio). The IHC method was followed as in our previous publication (19). IHC staining was examined with microscopy. IHC optical density scores were calculated to quantify protein expression in IHC images (20). CL-BCa samples were identified by the low expression of CLDN3/CLDN4/CLDN7/CDH1 in patients’ tissue biopsies. All patients were diagnosed and treated at The First Affiliated Hospital of Chongqing Medical University, and the informed consent was obtained from all patients.

## Results

### Key Modules of Gene Expression Were Identified in Claudin-Low BCa by WGCNA Analysis

**Figure 1:**
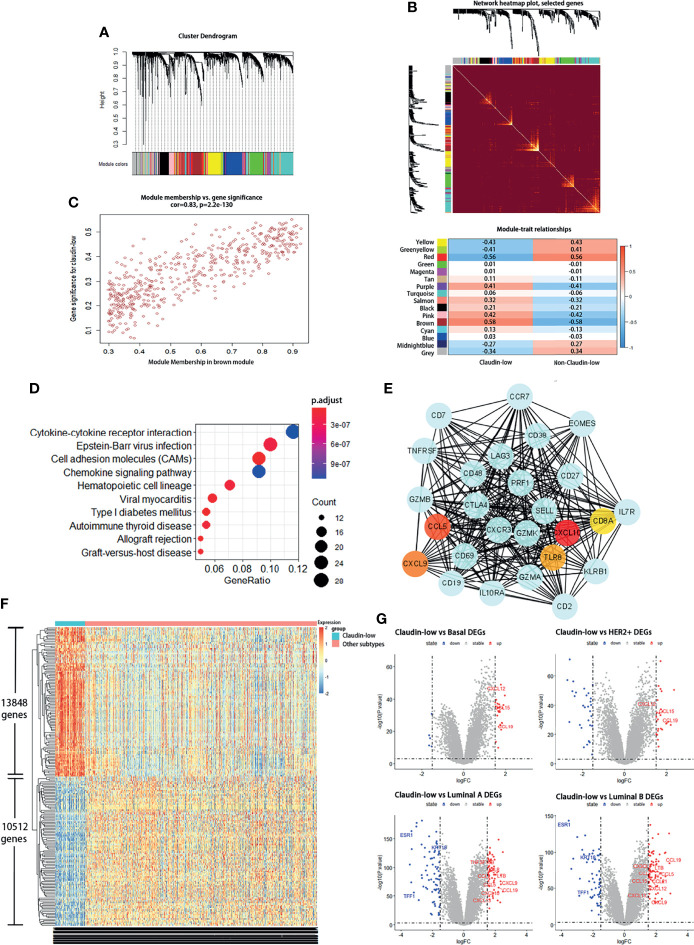
Claudin-low BCa exhibits differentially expressed genes and a distinct gene co-expression network by WGCNA. **(A, B)** Identification of gene co-expression networks in the claudin-low subtype of breast cancer *via* hierarchical average linkage clustering. **(C)** Relevant analysis between brown module and claudin-low breast cancer. Genes in the brown module showed higher co-expression interconnectedness. **(D)** KEGG analysis for genes in the brown module. **(E)** The top-scoring module of the PPI network detected by the MCODE; hub genes were marked with colors. **(F)** Heatmap plot showing different gene expressions between claudin-low and other subtypes. Red for upregulated genes and blue for downregulated genes. **(G)** Volcano plots for DEGs between claudin-low and other four subtypes of breast cancer. Red dots for upregulated genes and blue dots for downregulated genes.

To identify the modules of gene expression in claudin-low BCa using the WGCNA package in R, patients in METABRIC were divided into two groups, claudin-low (CL) group (199 cases) and non-claudin-low (NCL) group (1,559 cases). The top 5,000 genes with similar patterns of expression were categorized into modules *via* hierarchical average linkage clustering. Totally, 15 modules were identified between the CL group and NCL group and the relevance of each module was calculated (**Figures 1A, B**). We found that the brown module was the most relevant module to the trait of the claudin-low subtype (Cor = 0.83, p = 2.2e-130) (**Figure 1B**). Hub genes of the brown module also tend to be highly correlated with weight, which suggested that these hub genes might be the featured genes of claudin-low subtype (**Figure 1C**). Genes identified in the brown module belonged to gene sets associated with cytokine interaction (**Figure 1D**). The top 200 genes ranked by co-expressing correlation in the brown module were chosen to perform PPI network (protein–protein interaction network) construction using the STRING online database. The top-scoring module in the PPI network (score: 22.074) was analyzed using MCODE in Cytoscape software. Specially, we found that the hub genes in this module were CXCL9, CXCL10, CCL5, TLR8, CD8A and EOMES, which were relevant to the immune pathway and were detected by CytoHubba (**Figure 1E**).

To uncover the characteristics of claudin-low BCa, differentially expressed genes (DEGs) were used to portray the difference at the transcription level between CL-BCa and other subtypes of BCa. We found that the overall gene expression patterns at the mRNA level were significantly distinct between claudin-low and other subtypes (**Figure 1F**). Compared to the basal subtype, 67 DEGs were downregulated and 180 DEGs were upregulated in the claudin-low subtype. Relative to the HER2+ subtype, 333 DEGs were identified in claudin-low subtype and 204 DEGs were upregulated. Additionally, 515 and 584 differently expressed genes were identified in the claudin-low subtype compared with luminal A or luminal B, respectively. Since the genes identified in the brown module were enriched in the pathway associated with cytokine interaction (**Figure 1D**), we further investigated the cytokines which were upregulated in the claudin-low group by intersecting upregulated DEGs with the gene sets of cytokine-associated pathways. Compared with the basal or HER2+ subtype and using the 1.5 fold change as the threshold, three cytokines upregulated in claudin-low were identified (CXCL12, CCL15, CCL19) (**Figure 1G**). Compared with the luminal A subtype, nine cytokines (TNFSF13B, CCL8, LTB, CCL2, CCL5, CXCL9, CCL19, CXCL10, CXCL13) were upregulated in claudin-low; similarly, we found 10 cytokines in claudin-low compared to the luminal B subtype (CCL19, CX3CL1, LTB, CCL2, CCL5, CCL21, CXCL12, CCL15, CXCL13, CXCL9) (**Figure 1G**). These findings indicated that the brown module in WGCNA analysis was the most relevant to CL-BCa subtype and cytokines genes were remarkably DEGs in CL-BCa.

### Featured DEGs and Their Associated Pathways in Claudin-Low BCa

**Figure 2:**
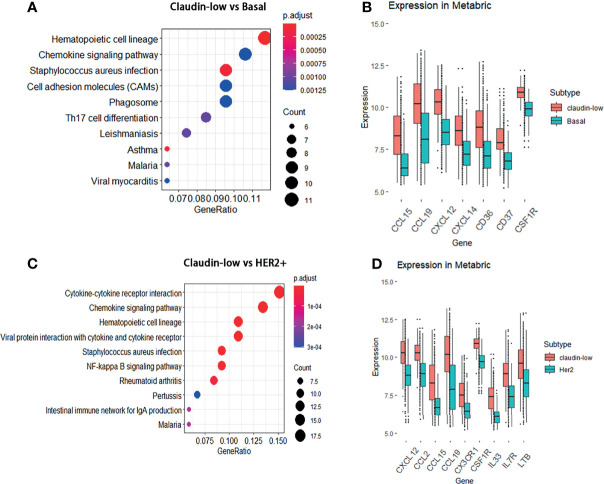
DEGs between the claudin-low and basal or HER2+ subtype and their involved pathways by KEGG analyses. **(A)** Dot plot of the upregulated genes involving pathways in the claudin-low subtype compared with the basal subtype. **(B)** Compared to the basal subtype, the significantly upregulated genes in claudin-low subtype were CCL15, CCL19, CXCL12, CXCL14, CD36, CD37 and CSF1R. **(C)** Dot plot of the upregulated genes involving pathways in the claudin-low compared with the HER2+ subtype. **(D)** Compared to the HER2+ subtype, the significantly upregulated genes in the claudin-low subtype were CXCL12, CCL2, CCL15, CCL19, CX3CR1, IL33, IL7R, LTB and CSF1R.

**Figure 3:**
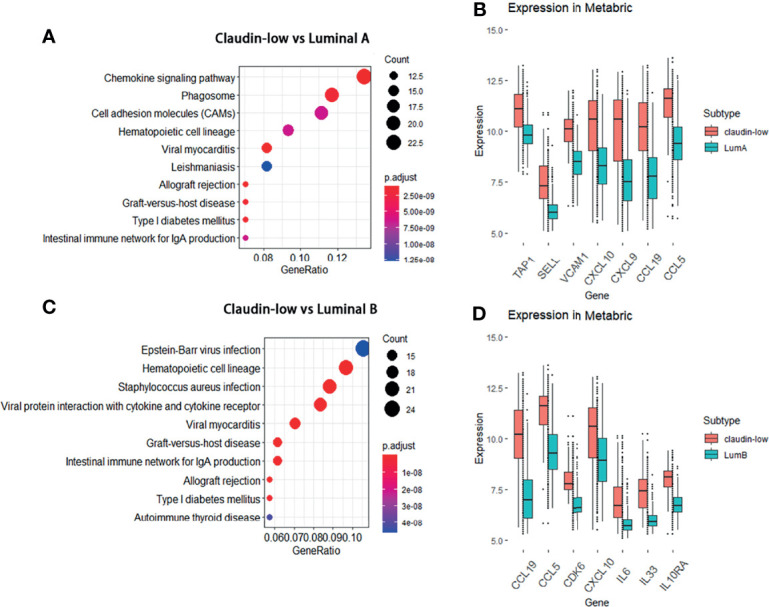
DEGs between the claudin-low and luminal A or luminal B subtype and their involved pathways by KEGG analyses. **(A)** Dot plot of the upregulated genes involving pathways in the claudin-low comparing with luminal A subtype. **(B)** Compared to the luminal A subtype, the significantly upregulated genes in claudin-low subtype were TAP1, SELL, VCAM1, CXCL10, CXCL9, CCL19 and CCL5. **(C)** Dot plot of upregulated pathways for the claudin-low compared with luminal B; **(D)** Compared to the luminal B subtype, the significantly upregulated genes in the claudin-low subtype were CCL19, CCL5, CDK6, CXCL10, IL6, IL33 and IL10RA.

Since the result of the WGCNA analysis hinted that cytokines were distinctive feature genes of claudin-low BCa subtype, we intended to explore the signal pathways that these differentially expressed cytokines were involved. In order to portray the signal pathways of these DEGs in claudin-low BCa, enrichment analysis on the DEGs was performed to clarify DEG-related pathways in the claudin-low subtype of the METABIRC dataset. Generally, pathways of enriched DEGs in CL-BCa were mainly associated with cytokine signals and cell adhesion (**Figures 2**, **3** and **Tables 1**, **2**). Compared to the basal subtype, upregulated genes were mainly involved in pathways of hematopoietic lineage and chemokine signaling (**Figure 2A**). Especially, the CCL15, CCL19, CXCL12 and CXCL14 in chemokine signaling pathways were the most upregulated chemokine genes in the claudin-low subtype (**Figure 2B**). Relative to the HER2+ subtype, upregulated DEGs mainly focused on signaling pathways of cytokine–cytokine receptor interaction and chemokine (**Figure 2C**). Among these DEGs, CXCL12, CCL2, CCL15 and CCL19 were top upregulated chemokine genes, while CX3CR1, CSF1R, IL33, IL7R and LTB were the most enhanced genes on cytokine–cytokine receptor interaction (**Figure 2D**). Next, enrichment analysis on DEGs between the claudin-low and luminal A or luminal B subtype was also performed by METABRIC. Specifically, compared to the luminal A subtype, the DEGs in the claudin-low subtype were mostly enriched in the chemokine signaling pathway, phagosome, and cell adhesion (**Figure 3A**). As upregulated genes, CXCL10, CXCL9, CCL19, CCL5 TAP1, SELL and VCAM1 were enhanced in the claudin-low subtype (**Figure 3B**). Similarly, compared to the luminal B subtype, genes related to virus infection and viral protein with the cytokine and cytokine receptor were mostly enhanced (**Figure 3C**). For instance, CCL19, CCL5, CDK6, CXCL10, IL6, IL33 and IL10RA were upregulated in the claudin-low subtype (**Figure 3D**). Meanwhile, the expression of downregulated genes in claudin-low tissues versus the other four subtypes was also identified (**Supplementary Figures 1A–F**). Taken together, based on the result of the WGCNA analysis and featured DEGs and their associated pathways in CL-BCa, cytokine/chemokine signaling might play a pivotal role in claudin-low BCa.

**Table 1:** Up-regulated genes and their pathway in Claudin-low BCa compared with four BCa subtypes.

Subtype	Pathway	Up-regulated Genes
Basal	Hematopoietic cell lineage	CD2,CD36,CD37,CD3D,CD8A, CSF1R,HLA-DOA,HLA-DPA1, HLA-DQA1,HLA-DRA,IL7R
	Chemokine signaling pathway	CCL15,CCL19,CCL21,CX3CR1, CXCL12,CXCL14,DOCK2, GNG11,PRKCB,RAC2
	Cytokine-cytokine receptor interaction	CCL15,CCL19,CCL21,CSF1R, CX3CR1,CXCL12,CXCL14,IL33, IL7R,TGFBR2
	Staphylococcus aureus infection	C1QB,C1R,C3,CFD,CFH,HLA-DOA,HLA-DPA1,HLA-DQA1, HLA-DRA
	Cell adhesion molecules (CAMs)	CD2,CD8A,CLDN5,HLA-DOA, HLA-DPA1,HLA-DQA1, HLA-DRA,PECAM1,SELL
HER2+	Cytokine-cytokine receptor interaction	CCL13,CCL15,CCL19,CCL2, CCL21,CCL4,CCL5,CCL8,CSF1R, CX3CR1,CXCL12,CXCR4,IL33,IL6,IL7R,LTB, TGFBR2,TNFSF13B
	Chemokine signaling pathway	CCL13,CCL15,CCL19,CCL2, CCL21,CCL4,CCL5,CCL8,CX3CR1,CXCL12,CXCR4,DOCK2,GNG11,PRKCB,RAC2, WAS
	Hematopoietic cell lineage	CD14,CD2,CD36,CD37,CD3D, CD8A,CSF1R,HLA-DPA1,KIT HLA-DQA1,HLA-DRA,IL6,IL7R,
	Viral protein interaction with cytokine and cytokine receptor	CCL13,CCL15,CCL19,CCL2, CCL21,CCL4,CCL5,CCL8,CSF1R, CX3CR1,CXCL12,CXCR4,IL6
	Human T-cell leukemia virus 1 infection	CCND2,CD3D,EGR1,EGR2,FOS,HLA-B,HLA-DPA1,HLA-DQA1,HLA-DRA,IL6,MMP7,MYC, TGFBR2
Luminal A	Chemokine signaling pathway	CCL13,CCL19,CCL2,CCL21,CCL4,CCL5,CCL8,CCR7,CX3CL1, CXCL10,CXCL13,CXCL9,CXCR4,CXCR5,DOCK2,FGR,ITK,LYN,NCF1,PRKCB,RAC2,STAT1,WAS
	Cytokine-cytokine receptor interaction	CCL13,CCL19,CCL2,CCL21,CCL4,CCL5,CCL8,CCR7,CD27, CX3CL1,CXCL10,CXCL13,CXCL9,CXCR4,CXCR5,IL10RA,IL32, IL7R,LTB,TNFRSF17,TNFRSF21,TNFSF13B
	Phagosome	C1R,C3,CD14,CD36,CORO1A,CYBA,CYBB,HLA-B,HLA-DMB, HLA-DOA,HLA-DOB,HLA-DPA1,HLA-DQA1,HLA-DRA,HLA-E,HLA-F,ITGB2,NCF1,NCF4,TAP1
	Epstein-Barr virus infection	CD19,CD247,CD3D,CDK6,CXCL10,HLA-B,HLA-DMB,STAT1,HLA-DOA,HLA-DOB,HLA-DPA1,HLA-DQA1,HLA-DRA,HLA-E,HLA-F,LYN,PLCG2,RUNX3,TAP1, TNFAIP3
	Cell adhesion molecules (CAMs)	CD2,CD6,CD86,CD8A,CDH3, HLA-B,HLA-DMB,HLA-DOA, HLA-DOB,HLA-DPA1,HLA-DQA1,HLA-DRA,HLA-E,HLA-F,ICOS,ITGB2,ITGB7,SELL,VCAM1
Luminal B	Cytokine-cytokine receptor interaction	CCL13,CCL15,CCL19,CCL2, CCL21,CCL4,CCL5,CCL8,CCR7, CD27,CSF1R,CX3CL1,CXCL10, CXCL12,CXCL13,IL10RA,IL32, CXCL9,CXCR4,CXCR5,IL33,IL6, IL7R,LTB,TGFBR2,TNFRSF17, TNFRSF21,TNFSF13B
	Epstein-Barr virus infection	CCND2,CD19,CD247,CD3D, CDK6,CXCL10,HLA-B,VIM, HLA-DMA,HLA-DMB,HLA-DOA,HLA-DOB,HLA-DPA1,HLA-DPB1,HLA-DQA1,LYN,PLCG2, HLA-DRA,HLA-E,HLA-F,IL6, RUNX3,SYK,TNFAIP3
	Chemokine signaling pathway	CCL13,CCL15,CCL19,CCL2, CCL21,CCL4,CCL5,CCL8,CCR7, WAS,CX3CL1,CXCL10,CXCL12, CXCL13,CXCL9,CXCR4,CXCR5, DOCK2,FGR,ITK,LYN,PRKCB, RAC2,
	Hematopoietic cell lineage	CD14,CD19,CD2,CD36,CD37, CD38,CD3D,CD7,CD8A,CSF1R, HLA-DMA,HLA-DMB,HLA-DOA,HLA-DOB,KIT,HLA-DPA1,IL6, HLA-DPB1,HLA-DQA1,IL7R, HLA-DRA, MME
	Staphylococcus aureus infection	C1QA,C1QB,C1QC,C1R,C1S,C3, CFH,DEFB1,HLA-DMA, HLA-DMB,HLA-DOA,HLA-DOB,HLA-DPA1,HLA-DPB1, HLA-DQA1,HLA-DRA,ITGB2, KRT14, KRT15,KRT17
	Cell adhesion molecules (CAMs)	CD2,CD6,CD8A,CDH3,HLA-B, HLA-DMA,HLA-DMB,HLA-DOA,HLA-DOB,HLA-DPA1, HLA-DPB1,HLA-DQA1,HLA-DRA,HLA-E,HLA-F,ICAM2,ITGB2, ITGB7, SELL,VCAM1

**Table 2:** Down-regulated genes and their pathway in Claudin-low BCa compared with four BCa subtypes.

Subtype	Pathway	Down-regulated genes
Basal	Cell cycle	CCNB2,CCNE1,CDC20, CDC45, MCM4,TTK
	Oocyte meiosis	CALML5,CCNB2,CCNE1, CDC20
	p53 signaling pathway	CCNB2,CCNE1,SERPINB5
	Cellular senescence	CALML5,CCNB2,CCNE1, FOXM1
	Cell adhesion molecules (CAMs)	CDH3,CLDN3,VTCN1
HER2+	Tight junction	CLDN3,CLDN7,CRB3,ERBB2, SLC9A3R1,TJP3
	Cell adhesion molecules (CAMs)	CDH1,CLDN3,CLDN7,CNTNAP2,SDC1
	PI3K-Akt signaling pathway	CREB3L4,ERBB2,ERBB3, FGFR4, MYB
Luminal A	Estrogen signaling pathway	ADCY1,CREB3L4,ESR1,KRT18, KRT19,PGR,RARA,TFF1
	PI3K-Akt signaling pathway	CCND1,CHAD,COL4A5, CREB3L4,ERBB3,FGFR3, IGF1R,MYB
	Focal adhesion	CCND1,CHAD,COL4A5, FLNB, IGF1R,VAV3
	Proteoglycans in cancer	CCND1,ERBB3,ESR1,FLNB, IGF1R,VAV3
Luminal B	Estrogen signaling pathway	ADCY1,CREB3L4,ESR1,FKBP4, KRT18,KRT19,RARA,TFF1
	PI3K-Akt signaling pathway	CCND1,CREB3L4,ERBB3, FGFR3, IGF1R,MYB
	AMPK signaling pathway	CCND1,CREB3L4,FBP1,IGF1R, SREBF1
	Tight junction	CCND1,CGN,MARVELD2, SLC9A3R1,TJP3

**Figure 4:**
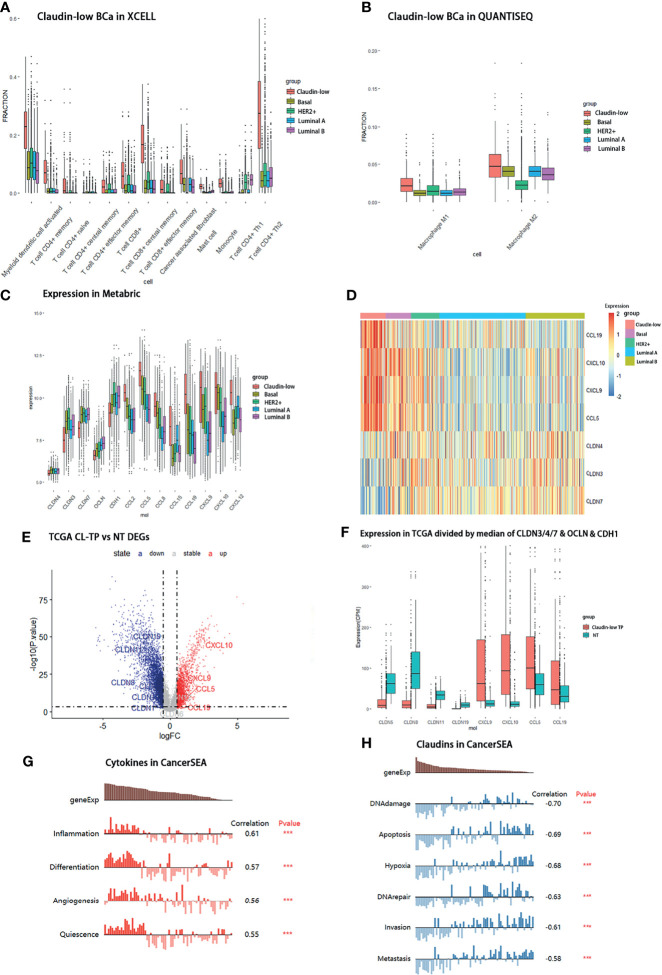
Distribution of immune cells and differential expression of claudin proteins and cytokines in claudin-low BCa. **(A)** Composition of immune cells in claudin-low, basal, and HER2+ groups assessed by QUANTISEQ. **(B)** Distribution of macrophages in claudin-low, basal, and HER2+ groups assessed by XCELL. **(C)** Different expression levels of cytokines in claudin-low and other subtypes in the METABRIC dataset. **(D)** Heatmap for tissue expression of CCL5, CCL19, CXCL9 and CXCL10 in METABRIC. **(E)** Differentially expressed genes in claudin-low BCa tissue in the TCGA-BRCA dataset compared to normal tissue (CL-TP presents claudin-low BCa; NT presents para-carcinoma normal tissue in BCa). **(F)** The expression levels of featured cytokines and claudins in the CL-BCa cohort of TCGA-BRCA defined by the median of CLDN3/4/7, OCLN and CDH1. **(G, H)** Function prediction of different expressed cytokines and claudins by CANCERSEA (***p < 0.001).

The preliminary result in our study has clarified that mostly upregulated DEGs in the claudin-low subtype were related to the cytokine–cytokine receptor interaction and chemokine signaling pathway compared to other BCa subtypes. Considering various interactions of cytokine/chemokine with immune cells, we sought to clarify the composition of tumor-infiltrating immune cells in claudin-low and non-claudin-low BCa tissue using the R package of “immunedeconv”. Generally, the fraction of tumor-infiltrating immune cells in claudin-low BCa tissues was significantly enhanced relative to non-claudin-low BCa. We found that T cells in claudin-low BCa strikingly increased compared with other BCa subtypes, especially T cell CD4+ memory, T cell CD8+ central memory and T cell CD4+ Th2 (**Figure 4A**). Specifically, the fraction of these T cells displayed distinctive differences between claudin-low BCa tissue and basal/HER2+ tissue, which indicated that increased T cell infiltration was a trait for claudin-low BCa (**Figure 4A**). In addition, claudin-low BCa tissue retained a higher fraction of immune cells than luminal A/luminal B tissues, including T cell CD4+ memory, T cell CD4+ naïve, T cell central/effector memory and T cell CD4+ Th2 (**Figure 4A**). Further, we analyzed the composition of macrophage in claudin-low and non-claudin-low tissues assessed by XCELL (**Figure 4B**). Compared with the basal or HER2+ subtype, fractions of both M1 and M2 macrophage were higher in tissues of the claudin-low subtype, which suggested that macrophages were probably more engaged in claudin-low BCa development (**Figure 4B**). Similarly, compared with the luminal A or luminal B subtype, M1 and M2 macrophage also showed a higher fraction in the claudin-low subtype (**Figure 4B**). Based on these observations, CD8+ T cell, CD4+ T cell, and macrophages were the distinctively enriched immune cells in claudin-low BCa.

### Expression Levels of Featured Cytokines and Claudins in METABRIC and Other BCa Databases

In order to verify the enriched expression of featured DEGs identified by KEGG, we examined the expression levels of featured DEGs (cytokines and claudins) in METABRIC and other BCa databases. Firstly, the expression levels of featured cytokines were examined in the METABRIC dataset. Seven differentially expressed cytokines (CCL15, CCL2, CCL19, CCL5, CXCL12, CXCL9 and CXCL10) had a higher expression level in the claudin-low subtype than non-claudin low subtypes (**Figures 4C, D**). Then, to confirm that these featured cytokines and claudins were also distinctively expressed in claudin-low BCa rather than in normal breast, we sought to screen the expression levels of these DEGs in the TCGA-BRCA dataset to detect the differences in their expression between claudin-low BCa tissue and normal breast tissue. Due to the lack of the claudin-low cohort in the TCGA-BRCA dataset, the claudin-low group was self-defined by dividing the TCGA-BRCA cohort according to the expression levels of CLDN3 and CLDN4 and CLDN7 and Occludin (OCLN) and E-cadherin (CDH1) (172 cases in the Claudin-low TP group and 113 cases in the normal group). We found that compared to the normal breast group, 6,270 downregulated genes and 4,770 upregulated genes were identified in the claudin-low BCa group (**Figure 4E**). Among these differentially expressed genes, CCL5, CCL19, CXCL9 and CXCL10 were upregulated in the claudin-low BCa group which were also identified as overexpressed genes in the METABRIC dataset; six claudin proteins (CLDN1, CLDN2, CLDN12, CLDN19, CLDN11 and CLDN8) were downregulated claudin proteins in the claudin-low BCa group compared to the normal group in addition to CLDN3&4&7 (**Figure 4F**). CLDN8, CLDN11 and CLDN19 were top three of the significantly downregulated claudins in our self-defined claudin-low group compared to the normal group (**Figure 4F**).

To further confirm the expression pattern of featured cytokines and claudins, we identified claudin-low patients in the Oslo2 database and TCGA dataset by the nine-cell line claudin-low predictor with the use of the R package “Genefu” (Oslo2 dataset: 13 CL-BCa cases and 352 non-CL-BCa cases; TCGA-BRCA: 33 CL-BCa cases and 1058 non-CL-BCa cases). Although the CL-BCa cases in the Oslo2 and TCGA databases were limited, CLDN11, CLDN19 and CLDN6 were downregulated claudins in CL-BCa compared to non-CL-BCa and CCL5, CXCL9 and CXCL10 were upregulated cytokines in CL-BCa compared to non-CL-BCa (**Supplementary Figures 2A, B**). Additionally, we investigated correlations between the expression of CLDN3/4/7 and cytokines (CCL5, CCL19 and CXCL9) expression in the GSE25066 dataset (**Supplementary Figure 4**). The negative correlations were shown between expressions of CLDN3/CLDN4/CLDN7 and CCL5/CCL19/CXCL9, which suggested that these upregulated cytokines (CCL5, CCL19 and CXCL9) could be used as potential biomarkers of CL-BCa patients with the feature of low-expressed CLDN3/CLDN4/CLDN7 (**Supplementary Figure 4**).

Finally, the potential functions of these cytokines and claudins in TCGA-BRCA were investigated by CANERSEA. These four cytokines were mostly correlated with inflammation, differentiation, and angiogenesis, which had an effect of promoting tumorigenesis (**Figure 4G**). It was worthwhile to mention that CCL5, CXCL9 and CXCL10 were the hub genes in the significant PPI module calculated by the DMNC algorithm, which indicated that these three cytokines probably played a crucial role in immune network (**Figure 1E**). The three featured claudin proteins were also negatively correlated with DNA damage, apoptosis, invasion, and metastasis (**Figure 4H**). Taken together, in claudin-low BCa, four cytokines (CCL5, CCL19, CXCL9 and CXCL10) were upregulated and three claudin proteins (CLDN8, CLDN11 and CLDN19) were downregulated, which were consequently selected as the genes of interest for the following analysis.

### Association Between Featured Cytokines in BCa Patients With Immune Cell Recruitment

**Figure 5:**
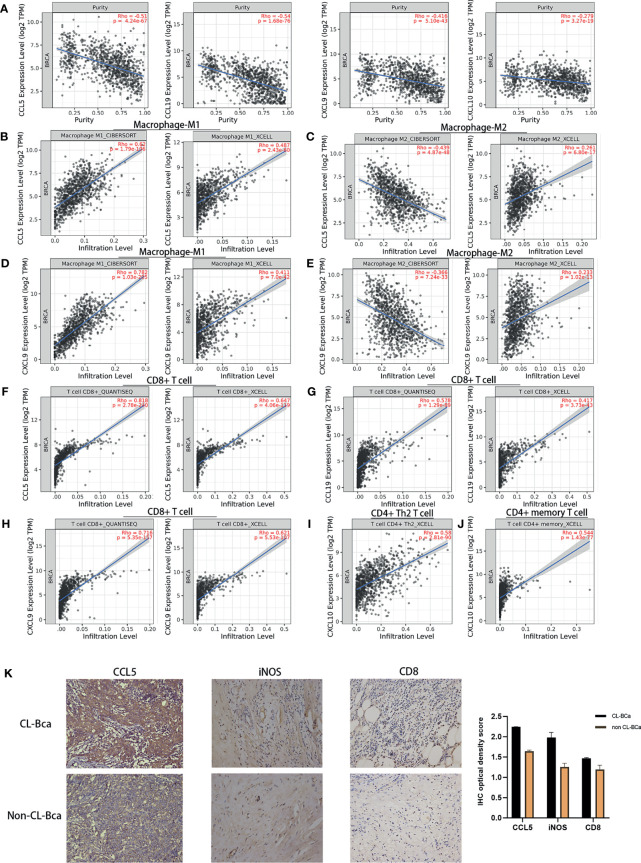
Expression levels of CCL5, CCL19, CXCL9, and CXCL10 in TCGA-BRCA patients correlated with immune cell recruitment. **(A)** The fraction of tumor cells negatively correlated with each of four featured cytokines (CCL5, CCL19, CXCL9, and CXCL10). The correlation between tumor purity and the expression of CCL5/CCL19/CXCL9/CXCL10 in TCGA-BRCA was shown. **(B)** Positive correlations between CCL5 expression and tumor-infiltrating M1 macrophage in TCGA-BRCA. **(C)** No positive correlations between CCL5 expression and tumor-infiltrating M2 macrophage. **(D)** Positive correlations between CXCL9 expression and tumor-infiltrating M1 macrophage. **(E)** No positive correlations between CXCL9 expression and tumor-infiltrating M2 macrophage. **(F)** Positive relationships between CCL5 expression and CD8+ T cell infiltration. **(G)** Positive relationships between CCL19 expression and CD8+ T cell infiltration. **(H)** Positive relationships between CXCL9 expression and CD8+ T cell infiltration. **(I)** Positive relationships between CXCL10 expression and CD4+ Th2 T cell infiltration. **(J)** Positive relationships between CXCL10 expression and CD4+ memory T cell infiltration. **(K)** High expression of CCL5, iNOS, and CD8 in CL-BCa tissues confirmed by IHC; the result of quantification of CCL5/iNOS/CD8 expression in IHC images is shown in the right part. More M1 macrophages and CD8+ T cells infiltrated in CCL5 highly expressed claudin-low BCa. Typical IHC images were shown.

To further investigate the relation of featured cytokine expression with immune cell recruitment, correlations of featured cytokines and immune cells were assessed by XCELL, CIBERSORT, and QUANTISEQ *via* TIMER2.0 in the BCa patient cohort of TCGA-BRCA. Firstly, we examined the correlation between each of the featured cytokines and tumor purity in TCGA-BRCA cohort which reflected the proportion of tumor cells in this sample cohort. The results of tumor purity showed that three cytokines (CCL5, CCL19 and CXCL9) were negatively correlated with tumor purity, which indicated that tumor cells were reduced but immune cells increased in tissues with high expression of these cytokines (**Figure 5A**).

Next, the results showed that there were positive correlations between CCL5 and M1 macrophage infiltration assessed by XCELL and CIBERSORT (Rho = 0.487 and 0.62, respectively) but no positive correlation between CCL5 and M2 macrophage by CIBERSORT (Rho = -0.366), which indicated that a high expression of CCL5 might facilitate infiltration of M1 macrophage into BCa tumor tissue (**Figures 5B, C**). Similar correlations were shown for CXCL9, which indicated that increased CXCL9 might enhance M1 macrophage infiltration into BCa tumor tissue (**Figures 5D, E**). Besides, CCL5, CCL19 and CXCL9 were positively correlated with CD8+ T cell infiltration assessed by QUANTISEQ (Rho = 0.818, 0.578, and 0.716, respectively), which suggested that a high expression of CCL5, CCL19, and CXCL9 might promote the tumor infiltration of CD8+ T cells in CL-BCa (**Figures 5F–H**). Finally, CXCL10 was positively correlated with CD4+ T cells, especially Th2 CD4+ T cell (Rho = 0.58) and memory CD4+ T cell (Rho = 0.544), which indicated that CXCL10 was associated with CD4+ T cell infiltration (**Figures 5I, J**).

To further confirm the connection between immune cells (CD8+ T cell and M1 macrophage) and cytokines in claudin-low BCa, we analyzed immunohistochemistry staining images from BCa. Among these featured cytokines, the expression of CCL5 was more relevant to the expression level of the M1 macrophage marker (iNOS) and CD8+ T cell marker (CD8). IHC results showed that a higher expression of iNOS and CD8 was observed in CCL5 highly expressed CL-BCa tissues, compared to non-CL-BCa tissues with a low CCL5 expression (**Figure 5K**). The results indicated that more M1 macrophages and CD8+ T cells existed in CL-BCa tissues with CCL5 high expression. In conclusion, we found that the protein expressions of CCL5, iNOS, and CD8 in CL-BCa tissues were higher than those in non-CL-BCa tissues and that more infiltration of M1 macrophages and CD8+ T cells was found in CCL5 highly expressed CL-BCa tissue. Taken together, the expression of these featured cytokines was associated with immune cell recruitment in CL-BCa tissues, which possibly altered the composition of immune cells in the tumor environment and thereby affected the prognosis of CL-BCa patients.

### Evaluation of Featured Cytokines and Claudin Proteins in Claudin-Low BCa for Prognosis

**Figure 6:**
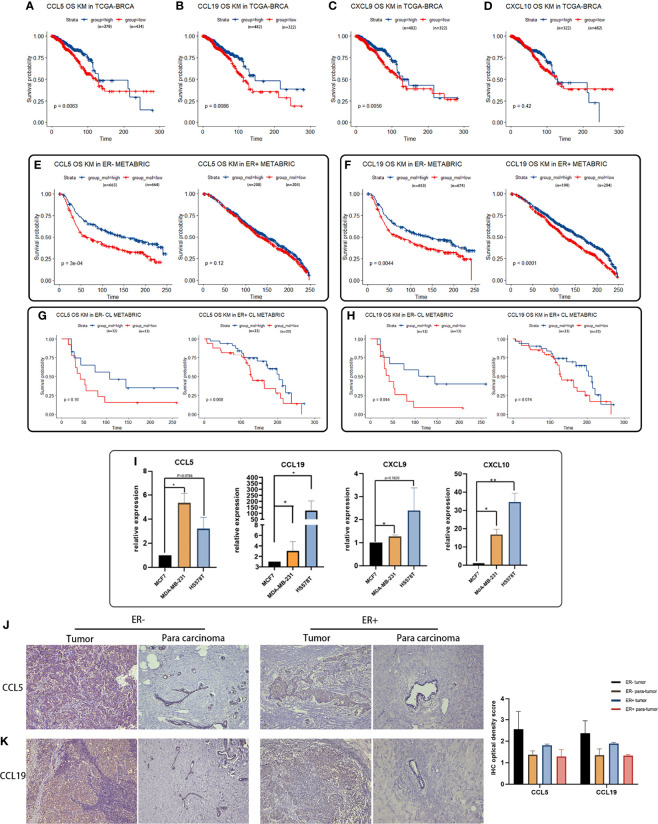
Survival analyses of featured cytokines and their expression in BCa cell lines and tissue. **(A–D)** Association between expression levels of CCL5 **(A)**, CCL19 **(B)**, CXCL9 **(C)**, and CXCL10 **(D)** in the TCGA-BRCA dataset with overall survival of BCa patients. High expression levels of CCL5, CCL19, and CXCL9 associated with better prognosis in breast cancer patient. **(E, F)** Expression of CCL5 **(E)** and CCL19 **(F)** in METABRIC associated with overall survival of BCa patients regarding of ER status. Highly expressed CCL5, CCL19, CXCL9, and CXCL10 were related to longer overall survival time in ER-negative BCa patients. **(G, H)** Association between expression of CCL5/CCL19 in METABRIC with overall survival of CL-BCa patients regarding of ER status. High expression of CCL5 **(G)** or CCL19 **(H)** was associated with longer overall survival of BCa patients in ER-negative CL-BCa patients. **(I)** High expression of CCL5, CCL19, CXCL9, and CXCL10 confirmed by qPCR in CL-BCa cell lines MDA-MB-231 and HS578T (ER-negative) (**p < 0.01; *p < 0.05, n = 3). **(J, K)** High expression of CCL5 and CCL19 confirmed by IHC in CL-BCa tissues. CCL5 **(J)** and CCL19 **(K)** were overexpressed in ER-negative tissues. Typical IHC results were shown (ER-negative BCa, n = 16; ER-positive BCa, n = 11), and the result of quantification of CCL5/CCL19 expression in IHC images is shown in the right part.

To clarify the relation between featured cytokines and claudin proteins with patients’ prognosis, Kaplan–Meier survival analysis was performed on the four upregulated cytokines (CCL5, CXCL9, CCL19 and CXCL10) and three downregulated claudin proteins (CLDN19, CLDN11 and CLDN8). Generally, high expression levels of CCL5, CCL19, CXCL9 and CXCL10 implied better prognosis, especially for the BCa patients with a negative ER status (**Figures 6A–H**). Firstly, expression levels of CCL5, CCL19, CXCL9 and CXCL10 were examined in TCGA-BRCA dataset for their relation with overall survival of BCa patients. Except CXCL10, high expression levels of CCL5, CCL19 and CXCL9 were significantly associated with better prognosis in breast cancer patients (**Figures 6A–D**). Additionally, multivariable Cox regression analyses were also performed in TCGA-BRCA dataset. A low expression of CCL5, CCL19 and CXCL9 was associated with poor outcome of BCa (**Supplementary Figure 5**).

Meanwhile, the relation between expressions of these cytokines and overall survival of BCa was also checked in the METABRIC dataset. Considering the lower expression of ER and ER-related genes in claudin-low BCa tissues than luminal tissues, we further investigated the association of these cytokines with overall survival of ER-positive BCa patients or ER-negative BCa patients. The result of **Figures 6E, F** indicated that patients with high expressions of CCL5 or CCL19 had a significantly longer overall survival in the cohort of ER-negative BCa patients rather than ER-positive BCa patients (**Figures 6E, F**). Moreover, we refined the claudin-low cohort in the METABRIC dataset by the nine-cell line claudin-low predictor and then evaluated the relation between CCL5/CCL19 with overall survival of CL-BCa patients (8, 15). A higher expression level of CCL5 was related with enhanced survival probability of ER-negative BCa patients, despite of the limited number of CL-BCa patients (23 ER-negative cases and 66 ER-positive cases) (**Figure 6G**). Likewise, higher CCL19 was also associated with better survival probability of ER-negative BCa patients (**Figure 6H**).

In addition, to further confirm the overexpression of these featured cytokines in claudin-low BCa, we detected their expression levels by qPCR in CL-BCa cell lines MDA-MB-231 and HS578T (ER-negative) and non-CL-BCa cell line MCF7 (ER-positive). Generally, the qPCR result indicated that these cytokines (CCL5, CXCL9, CCL19 and CXCL10) had a higher expression level in CL-BCa cell lines compared with the non-CL BCa cell line (**Figure 6I**). Specifically, compared to MCF7, the expression levels of CCL5, CXCL9, CCL19 and CXCL10 were significantly enhanced in MDA-MB-231, while the expression levels of CCL19 and CXCL10 were significantly raised in HS578T (**Figure 6I**). Finally, to further confirm the overexpression of CCL5 and CCL19 in BCa patients, we also examined the expression of CCL5 and CCL19 by IHC in both ER-positive BCa tissues and ER-negative CL-BCa tissues. Compared to in ER-positive BCa tissue, a higher expression of CCL5 and CCL19 was identified in ER-negative CL-BCa tissue, which was consistent with their expression in cell lines (**Figures 6J, K**). Therefore, regarding their significantly overexpression in CL-BCa, these featured cytokines had the potential as the prognosis markers for CL-BCa.

**Figure 7:**
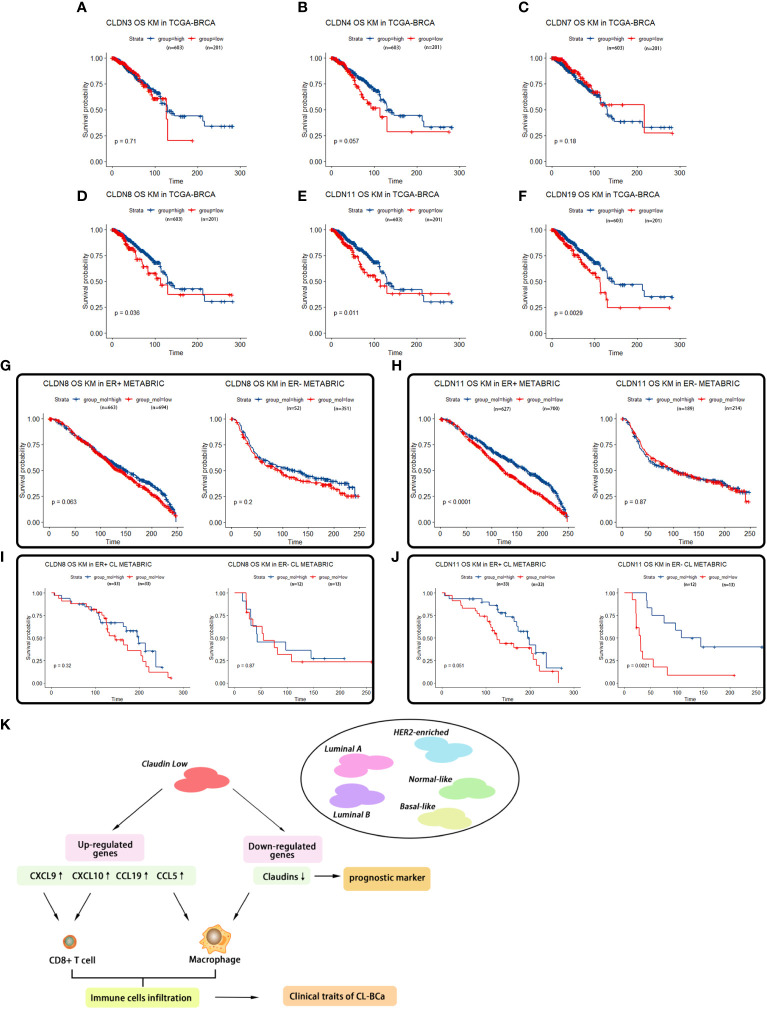
Survival analyses of featured claudins. **(A–C)** Expression levels of CLDN3/4/7 were not associated with overall survival time of BCa patients. No significant association between CLDN3 **(A)**, CLDN4 **(B)**, or CLDN7 **(C)** with overall survival of BCa patients in TCGA-BRCA. **(D–F)** Low expression levels of CLDN19/11/8 were associated with shorter overall survival of BCa patients, respectively. Significant association between CLDN19 **(D)**, CLDN11 **(E)**, or CLDN8 **(F)** and overall survival of BCa patients in the TCGA-BRCA dataset. **(G, H)** Association of expression of CLDN8 or CLDN11 in the METABRIC dataset with overall survival of BCa patients regarding ER status. Low expression of CLDN8 **(G)** or CLDN 11 **(H)** was related with shorter overall survival of BCa patients in ER-positive BCa patients. **(I, J)** Association of expression of CLDN8 or CLDN11 with overall survival of CL-BCa patients regarding of ER status. CLDN8 **(I)** had no significant effect on overall survival of CL-BCa patients. Low expression of CLDN 11 **(J)** was associated with shorter overall survival of BCa patients in ER-negative CL-BCa patients. **(K)** Workflow of the analytical process in this work.

A Kaplan–Meier survival analysis on featured claudin proteins was also performed in TCGA-BRCA and METABRIC datasets. In TCGA-BRCA dataset, unfortunately, we found that none of CLDN3, CLDN4 and CLDN7 showed a statistical relationship with survival of BCa patients, which suggested that CLDN 3/4/7 has no potential to be used as a predictive marker for overall survival (**Figures 7A–C**). However, a low expression of CLDN8, CLDN11 and CLDN19 was significantly related to shorter survival of BCa patients (**Figures 7D–F**), which was consistent with their lower expression levels in claudin-low BCa tissues (**Figure 4F**). Further, multivariable Cox regression analyses were also performed in TCGA-BRCA dataset. A low expression of CLDN8, CLDN11 and CLDN19 was related to poor outcome of BCa (**Supplementary Figure 5**). In the METABRIC dataset, for the cohort of overall BCa patients, we surprisingly found that low-expressed CLDN11 was related to shorter overall survival in ER-positive BCa patients, but there was no significant difference in ER-negative patients (**Figures 7G, H**). By evaluating the association of CLDN8 and CLDN11 with overall survival in the refined claudin low cohort in METABRIC, we found that patients with low expression of CLDN8 or CLDN11 showed shorter overall survival time; however, low expression of CLDN11 was more significantly associated with worse overall survival in CL-BCa patients (**Figures 7I, J**). Combining the results of the survival analysis and expression levels of these claudins in databases, CLDN8 and CLDN11 may be the featured members of the claudin family which were associated with the overall survival of CL-BCa, rather than CLDN 3&4&7. However, more studies are needed to explore the specific function of these featured claudins in BCa.

## Discussion

Claudin-low BCa is considered as the most primitive breast malignancy and remains poorly understood. Although some characteristics of gene expression in claudin-low BCa have been studied, specific genes associated with infiltration of immune cells in CL-BCa were still not clarified clearly. Therefore, Claudin-low breast cancer has aroused more attention to detect its clinical characteristics. This subtype is characterized by a low expression of claudin 3&4&7, and these claudin proteins are crucial for endothelial cells to form a substantial barrier between tumor tissue and blood vessel (8, 9). Despite of the varied factors affecting tumor metastasis, there are key factors such as the interaction and communication of tumor cells with their surrounding lymphocytes or tumor cells, including the activation of tumor cell chemotaxis through a specific chemoattractant in a cell–cell or cell–matrix cross talk (21, 22). Chemokine, as a type of secreted small cytokine or signaling protein, can serve as a guide molecule and direct the migration of immune cells resulting in changes of the microenvironment of tumor tissue and increasing the possibility of tumor metastasis (23–25). In our study, we found four differentially expressed cytokines/chemokines (CCL5, CCL19, CXCL9 and CXCL10) that regulated lymphocyte recruitment into tumor tissue and all of them were upregulated in claudin-low BCa (vs. other BCa subtypes) by the METABRIC database and in primary breast cancer patients defined as claudin-low BCa (vs. normal patients) by the TCGA-BRCA database. Additionally, the overexpression of these chemokines was also confirmed in CL-BCa cell lines and tissues by qPCR and IHC (**Figure 6**). Among these cytokines/chemokines, three of them belonged to the aspects of the CCL5-CCR5 axis, CCL19-CCR7 axis, and CXCL9/10-CXCR3 axis and their function played a crucial role in claudin-low BCa regarding their differential expression relative to other BCa subtypes.

CCL5, as a ligand of several chemokine receptors, binds to CCR1/3/5 and acts as a chemoattractant for blood monocytes, memory T-helper cells and eosinophils, which is involved in immunoregulatory and inflammatory processes (26). The CCL5–CCR5 pathway is one of the most important chemoattractant signal axes in BCa and increased CCL5 expression in tumor tissue is largely correlated with an advanced stage of breast cancer (27, 28). The CCL5/CCR5 axis is the main player in tumor progression, upregulated CCL5 in BCa tissue is one of the factors for facilitating the recruitment and infiltration of macrophages into BCa tissue (29) and recruitment of macrophages into the tumor microenvironment may promote tumor progression (27). Moreover, CCL5 also functions as a pro-tumor effector by promoting cancer cell proliferation, survival, motility, epithelial–mesenchymal transition (EMT) and stemness maintenance (27, 28). The upregulated expression of CCL5 and increased macrophages were both observed in CL-BCa; thus, we speculated that CCL5 might be one of the reasons for the increased recruitment of macrophage (30–32).

CXC-chemokines ligand 9 (CXCL9) and CXCL10 are the ligands of CXC-chemokine receptor 3(CXCR3) located on the membrane of effector CD8+ T cells. CXCL9 and CXCL10 are involved in CD8+ T cells infiltrating into tumor tissues (33). As expected, we observed an increased ratio of CD8+ T cells infiltrating into tumor tissues in claudin-low BCa compared with other subtypes (**Figure 4A**). It has also been reported that increased levels of CXCL9/10 were associated with increased numbers of tumor-infiltrating CD8+ T cells and CXCL9/10 were related with efficiency of immune therapy by blocking PD-L1 and PD-1 interaction (34). Here, we revealed that a strikingly high expression of CXCL9 was observed in claudin-low BCa and their expression was correlated with CD8+ T cell infiltration (**Figure 5H**). Therefore, CXCL9 might play an important role in CD8+ T cell-mediated immune response in claudin-low BCa.

CCL19 is one of the ligands of C–C chemokine receptor 7 (CCR7) which is a G protein-coupled receptor (GPCR) and commonly expressed by various lymphocyte cells, including naive T cells, central memory T cells, regulatory T cells, B cells and NK cells (35). The interaction of CCL9 with membrane-associated CCR7 is involved in an active chemotactic response of immune cells, including macrophages and T lymphocytes. It was reported that antibody-mediated neutralization of CCL19 in newborn mice impaired the thymic emigration of T cells into the peripheral circulation (36). Thus, a high level of secreted CCL19 in claudin-low BCa tissue contributed to the T cell emigration resulting in T cell infiltration. Additionally, the stimulation by CCL19 or CCL21 led to overexpression of CCR7 on the tumor cells and promoted cancer cell migration and infiltration into lymph nodes, which have been confirmed by the observation that increased CCR7 on cancer cell enhanced metastasis from breast to lung (37).

In addition, these upregulated cytokines/chemokines were associated with the overall survival of BCa patients by recruitment of immune cells. Through Kaplan–Meier survival analysis, we found that four cytokines (CCL5, CCL19, CXCL9 and CXCL10) were positively associated with the overall survival (OS) of BCa patients, especially ER-negative patients (**Figures 6E, F**). A recent study by Dangaj revealed that induced CCL5 and CXCL9 by macrophages could enhance the infiltration of immunoreactive CD8+ T cells into ovarian tumor and promote the overall survival of patients (38). This observation was consistent with our result that the ratio of CD8+ T cells and macrophage was higher in claudin-low BCa relative to other subtypes (**Figures 4A, B**). Thus, upregulated chemokines could act as predictive markers for overall survival.

As key integral tight junction proteins, although CLDN3/4/7 were featured claudins that were downregulated in CL-BCa, their prognosis value was limited. A recent study by Fougner characterized a claudin-low subtype by a self-defined method (39). However, information about differentially expressed claudin proteins associated with overall survival is still unclear. In this study, we found that CLDN11 and CLDN8 were downregulated in claudin-low BCa. Especially, CLDN11 was positively regulated by the transcription factor GATA family at the transcription level (40). Combining with our observation that a low expression level of CLDN11 was associated with short overall survival of BCa patients and that GATA3 was depressed in CL-BCa compared with ER-positive Luminal A and Luminal B BCa (**Supplementary Figures 1E, F**), downregulated GATA3 in CL-BCa was probably responsible for the decrease in CLDN11 expression. Furthermore, we found that CLDN11 and CLDN19 were negatively associated with infiltration of macrophage in claudin-low BCa tissue. Specifically, the expression of CLDN11 was negatively correlated with M1 macrophage (Rho = -0.314, p = 3.05e-24) assessed by XCELL in TCGA database (**Supplementary Figure 3A**). Taken together, CLDN11 which served as a hub gene associated with immune cell recruitment in claudin-low BCa could act as a potential marker of poor prognosis.

The interplay between extracellular molecules (e.g., cytokine/chemokine) and cellular receptors and the interactions between cell adhesion molecules on neighboring cells remain unclear. The tumor microenvironment is a complicated system that involves multiple players, and both chemokines and their receptors exhibit multiple functions in regulating immune response. Based on the results of tumor-infiltrating immune cells calculated by the enrichment of immune cell signatures and IHC staining in the BCa tissues, we drew a preliminary conclusion that there were more tumor-infiltrating immune cells in the claudin-low BCa subtype than the other subtypes and that increased cytokines/chemokines (CCL5, CCL19, CXCL9, and CXCL10) mediating chemotaxis definitely played a predominant role in this process, which contributed to understanding the features of claudin-low BCa and developing a potential strategy for claudin-low BCa patients.

## Conclusions

In summary, we specifically revealed the gene expression signature of chemokines/cytokines and the relationships between chemokine expression and infiltration of immune cells in claudin-low BCa (**Figure 7K**). As featured DEGs, CCL5, CCL19, CXCL9 and CXCL10 were overexpressed in claudin-low breast cancer cells and tissues which were positively correlated with tumor-infiltrating immune cells, especially CD8+T cells and macrophages, but CLDN8 and CLDN11 were repressed in claudin-low breast cancer cells and tissues. These featured cytokines (CCL5, CCL19 and CXCL9) and claudin proteins (CLDN8, CLDN11 and CLDN19) were related to BCa patients’ overall survival. Actually, there is no exact marker to identify claudin-low BCa in the routine clinical workup and the cases could therefore be probably missed. Our analyses are working out a path to identify markers and approaches to better stratify claudin-low breast cancer. These featured chemokines have potential as prognostic biomarkers and therapeutic targets to promote diagnostic and immunotherapy sensitivity for improving outcomes of breast cancer patients. Finally, our finding on immunological characteristics in the TME of CL-BCa will contribute to the understanding of claudin-low BCa.

## Data Availability

The original contributions presented in the study are included in the article/**Supplementary Material**. Further inquiries can be directed to the corresponding authors.
