# Corrigendum: Identification of differentially expressed hub genes associated with immune cell recruitment in claudin-low breast cancer

**DOI:** 10.3389/fonc.2023.1335637

**Published:** 2024-01-04

**Authors:** Yange Wang, He Shi, Yulu Zhang, Qian Zeng, Tingmei Chen, Chengsen Chai

**Affiliations:** Key Laboratory of Clinical Laboratory Diagnostics (Ministry of Education), College of Laboratory Medicine, Chongqing Medical University, Chongqing, China

**Keywords:** Claudin-low breast cancer, differentially expressed genes, cytokine, chemokine, tumor-infiltrating immune cell

In the published article, there was an error in **Figure 7** as published. Unfortunately, the data from recently replicated experiments suggests that the results in **Figures 7K-L** are not sufficiently solid for publication. Therefore, **Figures 7K-L** in **Figure 7** has been amended; **Figure 7M** has been relabeled to **Figure 7K**.

The corrected **Figure 7** and its caption “*Survival analyses of featured claudins”* appear below.

**Figure 7:**
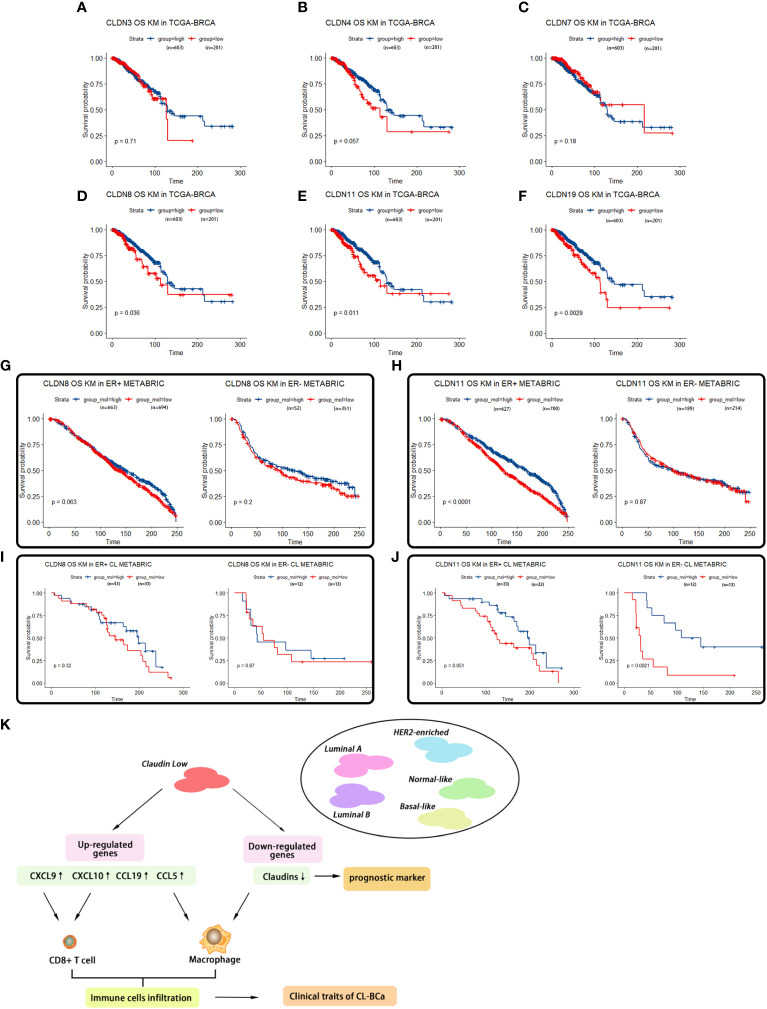
Survival analyses of featured claudins. **(A–C)** Expression levels of CLDN3/4/7 were not associated with overall survival time of BCa patients. No significant association between CLDN3 **(A)**, CLDN4 **(B)**, or CLDN7 **(C)** with overall survival of BCa patients in TCGA-BRCA. **(D–F)** Low expression levels of CLDN19/11/8 were associated with shorter overall survival of BCa patients, respectively. Significant association between CLDN19 **(D)**, CLDN11 **(E)**, or CLDN8 **(F)** and overall survival of BCa patients in the TCGA-BRCA dataset. **(G, H)** Association of expression of CLDN8 or CLDN11 in the METABRIC dataset with overall survival of BCa patients regarding ER status. Low expression of CLDN8 **(G)** or CLDN 11 **(H)** was related with shorter overall survival of BCa patients in ER-positive BCa patients. **(I, J)** Association of expression of CLDN8 or CLDN11 with overall survival of CL-BCa patients regarding of ER status. CLDN8 **(I)** had no significant effect on overall survival of CL-BCa patients. Low expression of CLDN 11 **(J)** was associated with shorter overall survival of BCa patients in ER-negative CL-BCa patients. **(K)** Workflow of the analytical process in this work.

In the published article, there was an error. Descriptions of the experiments relating to Claudin-11 have been removed due to instability of previously conducted experiments. These changes are listed below.

A correction has been made to Abstract, Paragraph 1. This sentence previously stated:

“Differentially expressed cytokines (CCL5, CCL19, CXCL9 and CXCL10) and claudins (CLDN8, CLDN11 and CLDN19) were related to the overall survival, and their expression levels were also examined both in tumor tissues of CL-BCa patients by IHC and in typical CL BCa cell lines by qPCR. Finally, the BCa patients with high expression of these DEGs (CCL5, CCL19, CXCL9, CLDN8 and CLDN11) showed a better overall survival”.

The corrected sentence appears below:

“Differentially expressed cytokines (CCL5, CCL19, CXCL9 and CXCL10) were related to the overall survival, and their expression levels were also examined both in tumor tissues of CL-BCa patients by IHC and in typical CL BCa cell lines by qPCR. Moreover, the BCa patients with low expression of these differentially expressed claudins (CLDN8, CLDN11 and CLDN19) showed a worse overall survival”.

A correction has been made to Materials and Methods, *Quantitative Polymerase Chain Reaction*, Paragraph 3. This sentence previously stated:

“The sequences of qPCR primers used were presented as follows: CLDN11, 5′-TGGATTGGTGCTGTGCTGTGC-3′ (forward) and 5′-GAGCCCGCAGTGTAGTAGAAACG-3′ (reverse); CLDN8, 5′-CACTGGTGCTGATTGTTGGAGGAG-3′ (forward) and 5′-TTCTGGAGTAGACGCTCGGTGAC-3′ (reverse); CCL5, 5′-CAGCAGTCGTCCACAGGTCAAG-3′ (forward) and 5′-TTTCTTCTCTGGGTTGGCACACAC-3′ (reverse); CCL19, 5′-AGCCTGCTGGTTCTCTGGACTTC-3′ (forward) and 5′-AGGGATGGGTTTCTGGGTCACAG-3′ (reverse); CXCL9, 5′-TCTTGCTGGTTCTGATTGGAGTGC-3′ (forward) and 5′-GTCCCTTGGTTGGTGCTGATGC-3′ (reverse); CXCL10, 5′-AACTGTACGCTGTACCTGCATCAG-3′ (forward) and 5′-ACGTGGACAAAATTGGCTTGCAG-3′ (reverse)”.

The corrected sentence appears below:

“The sequences of qPCR primers used were presented as follows: CCL5, 5′-CAGCAGTCGTCCACAGGTCAAG-3′ (forward) and 5′-TTTCTTCTCTGGGTTGGCACACAC-3′ (reverse); CCL19, 5′-AGCCTGCTGGTTCTCTGGACTTC-3′ (forward) and 5′-AGGGATGGGTTTCTGGGTCACAG-3′ (reverse); CXCL9, 5′-TCTTGCTGGTTCTGATTGGAGTGC-3′ (forward) and 5′-GTCCCTTGGTTGGTGCTGATGC-3′ (reverse); CXCL10, 5′-AACTGTACGCTGTACCTGCATCAG-3′ (forward) and 5′-ACGTGGACAAAATTGGCTTGCAG-3′ (reverse)”.

A correction has been made to Materials And Methods, *Immunohistochemistry*, Paragraph 3. This sentence previously stated:

“The expression of DEGs (CCL5, CCL19, and CLDN11) in BCa patients was examined by immunohistochemistry (IHC). Antibodies used for IHC were CCL5 (Cat#abs136939, Absin, Shanghai, China), CCL19 (Cat#abs149041, Absin), CLDN11 (Cat#abs131464, Absin), iNOS (Cat#GB11119, Servicebio, Wuhan, China), and CD8 (Cat#GB13068, Servicebio)”.

The corrected sentence appears below:

“The expression of DEGs (CCL5 and CCL19) in BCa patients was examined by immunohistochemistry (IHC). Antibodies used for IHC were CCL5 (Cat#abs136939, Absin, Shanghai, China), CCL19 (Cat#abs149041, Absin), iNOS (Cat#GB11119, Servicebio, Wuhan, China), and CD8 (Cat#GB13068, Servicebio)”.

A correction has been made to Results, *Key Modules of Gene Expression Were Identified in Claudin-Low.*

*BCa by WGCNA Analysis*, Paragraph 3. This sentence previously stated:

“Compared with the luminal A subtype, nine cytokines (TNFSF13B, CCL8, LTB, CCL2, CCL5, CXCL9, CCL19, CXCL10, CXCL13) were upregulated in claudin-low; similarly, we found 10 cytokines in claudin-low compared to the luminal B subtype (CCL19, CX3CL1, LTB, CCL2, CCL5, CCL21, CSCL12, CCL15, CXCL13, CXCL9) (Figure 1G)”.

The corrected sentence appears below:

“Compared with the luminal A subtype, nine cytokines (TNFSF13B, CCL8, LTB, CCL2, CCL5, CXCL9, CCL19, CXCL10, CXCL13) were upregulated in claudin-low; similarly, we found 10 cytokines in claudin-low compared to the luminal B subtype (CCL19, CX3CL1, LTB, CCL2, CCL5, CCL21, CXCL12, CCL15, CXCL13, CXCL9) (Figure 1G)”.

A correction has been made to Results, *Evaluation of Featured Cytokines and Claudin Proteins in Claudin-Low BCa for Prognosis*, Paragraph 12. This sentence previously stated:

“However, when we evaluated the relation of CLDN8/11 with overall survival in the refined claudin low cohort in METABRIC, a lower expression of CLDN11 was significantly associated with worse overall survival (**Figures 7I, J**). These observations indicated that the relation between CLDN11 expression and survival probability of overall BCa patients was different with that in the CL-BCa subtype. Additionally, we also examined the expression levels of CLDN8 and CLDN11 in CL-BCa cell lines MDA-MB-231 and HS578T(ER-negative) and non-CL-BCa cell line MCF7 (ER-positive) by qPCR. Moreover, the qPCR results showed that compared to MCF7, the expression of CLDN11 was significantly depressed in both CL-BCa cell lines MDA-MB-231 and HS578T, while the expression of CLDN8 was decreased in MDA-MB-231 but dramatically enhanced in HS578T (**Figure 7K**). Finally, we also examined the expression of CLDN11 by IHC in both ER-positive and ER-negative CL-BCa tissues. Compared to ER-negative BCa tissue, a higher expression of CLDN11 was identified in ER positive BCa tissue, which was consistent with their expression in cell lines (**Figure 7L**). Therefore, combining the results of the survival analysis and expression levels of these claudins, CLDN8 and CLDN11 might show them to be featured members of the claudin family which were associated with the overall survival of CL-BCa instead of CLDN 3&4&7”.

The corrected sentence appears below:

“By evaluating the association of CLDN8 and CLDN11 with overall survival in the refined claudin low cohort in METABRIC, we found that patients with low expression of CLDN8 or CLDN11 showed shorter overall survival time; however, low expression of CLDN11 was more significantly associated with worse overall survival in CL-BCa patients (**Figures 7I, J**). Combining the results of the survival analysis and expression levels of these claudins in databases, CLDN8 and CLDN11 may be the featured members of the claudin family which were associated with the overall survival of CL-BCa, rather than CLDN 3&4&7. However, more studies are needed to explore the specific function of these featured claudins in BCa”.

A correction has been made to Conclusions, Paragraph 14. This sentence previously stated:

“In summary, we specifically revealed the gene expression signature of chemokines/cytokines and the relationships between chemokine expression and infiltration of immune cells in claudin-low BCa (**Figure 7M**)”.

The corrected sentence appears below:

“In summary, we specifically revealed the gene expression signature of chemokines/cytokines and the relationships between chemokine expression and infiltration of immune cells in claudin-low BCa (**Figure 7K**)”.

The authors apologize for this error and state that this does not change the scientific conclusions of the article in any way. The original article has been updated.

