# Supplementary material for: Identification of Differentially Expressed Hub Genes Associated With Immune Cell Recruitment in Claudin-Low Breast Cancer
Source: Front Oncol. 2022 Mar 11;12:848206. doi: 10.3389/fonc.2022.848206 (PMC8963482; doi:10.3389/fonc.2022.848206)
Supplement: Supplementary file 1 [file DataSheet_1.pdf]

Supplementary Material

1 Supplementary Figures and Tables

1.1 Supplementary Figures

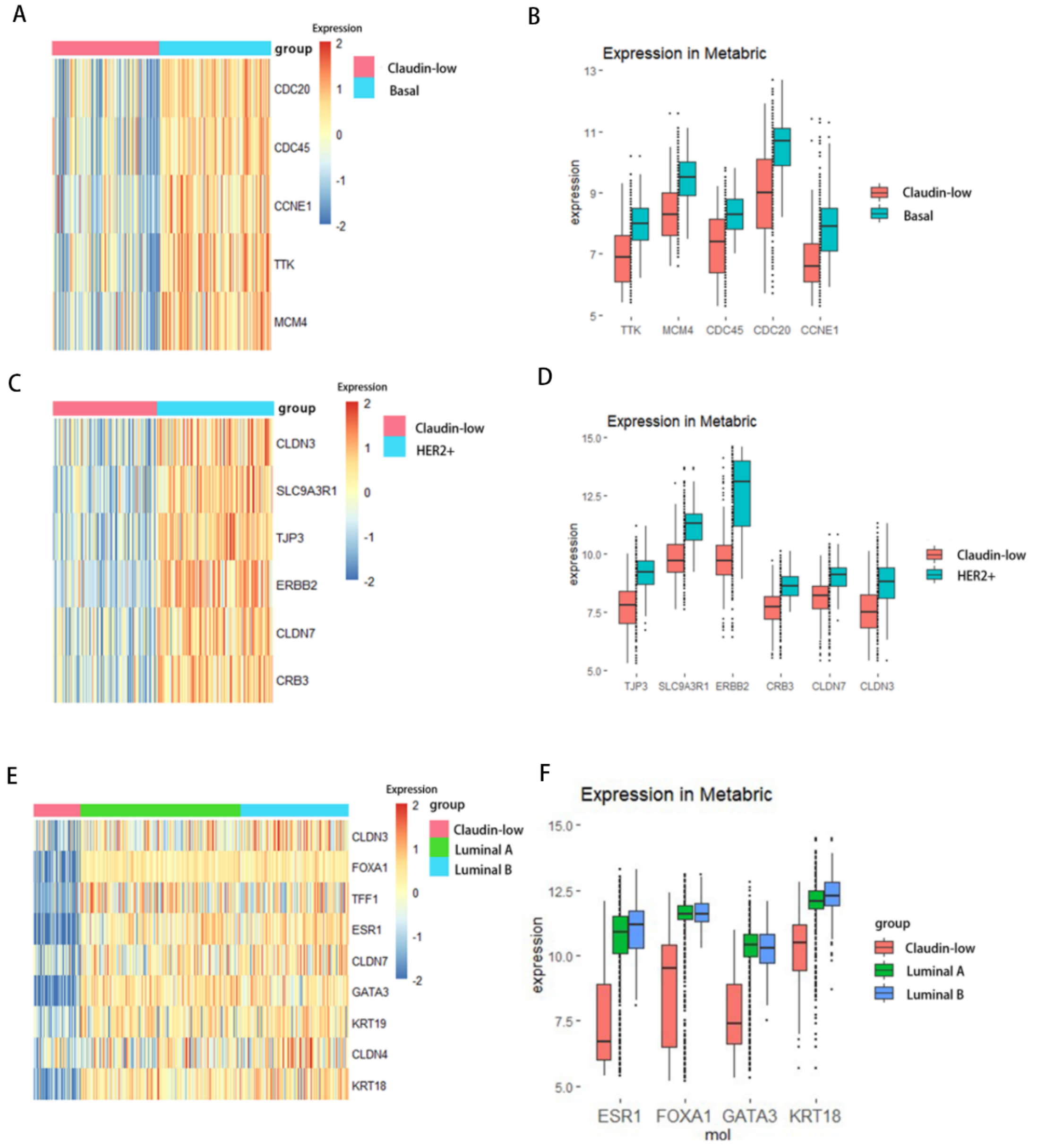

Supplementary Figure 1. Down-regulated genes in claudin-low BCa. (A) Tissue expression of five down-regulated DEGs (TTK, MCM4, CDC45, CDC20 and CCNE1) in comparison between claudin-low subtype and basal subtype. (B) Expression levels of six featured DEGs (ERBB2/HER2, CLDN3, CLDN7, CRB3, TJP3 and SLC9A3R1) between basal subtype and claudin-low subtype in

METABRIC. (C) Tissue expression of featured DEGs in comparison between claudin-low subtype and HER2+ subtype. (D) Expression levels of featured DEGs between HER2+ subtype and claudin-low subtype in METABRIC. (E) Expression of featured DEGs in BCa tissue (CLDN3/4/7 and ER-signal pathway associated molecules); (F) Expression levels of ESR1 & FOXA1 & GATA3 in luminal A, luminal B and claudin-low BCa subtypes.

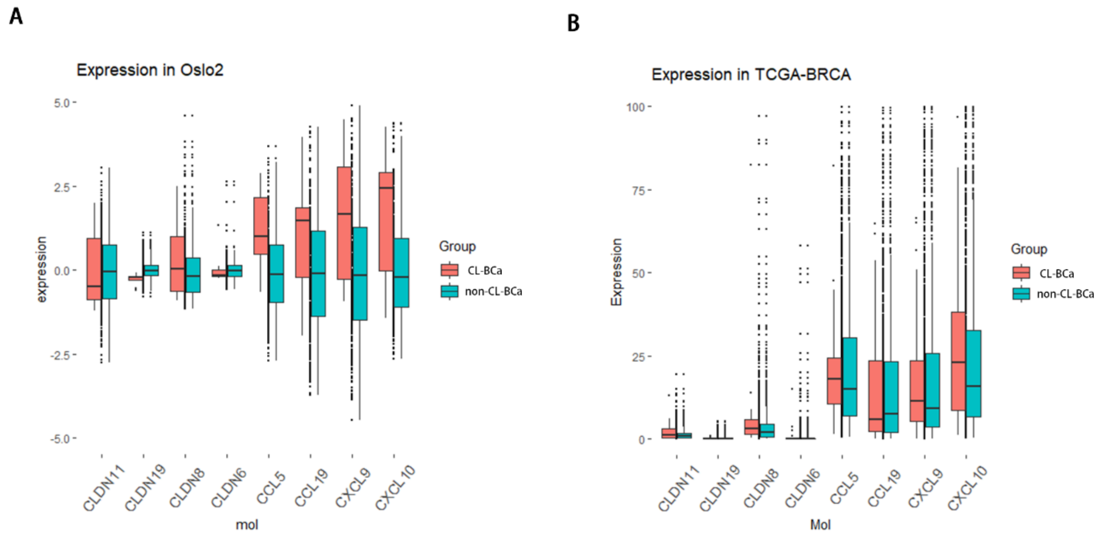

Supplementary Figure 2. Different expression levels of featured cytokines and claudins in CL-BCa cohort defined by nine-cell line claudin low predictor in Oslo2 dataset and TCGA-BRCA dataset (Oslo2 dataset: 13 CL-BCa cases and 352 non-CL-BCa cases; TCGA-BRCA: 33 CL-BCa cases and 1058 non-CL-BCa cases).

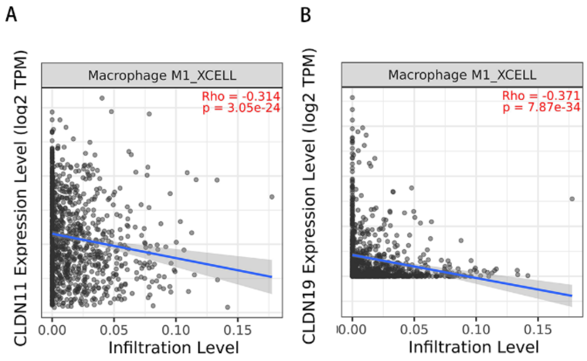

Supplementary Figure 3. Expression levels of CLDN11 and CLDN19 in TCGA-BRCA patients correlated with immune cell recruitment. (A-B) Negative correlations between CLDN11(A) and CLDN19(B) expression and tumor-infiltrating M1 macrophage assessed by XCELL in TCGA-BRCA.

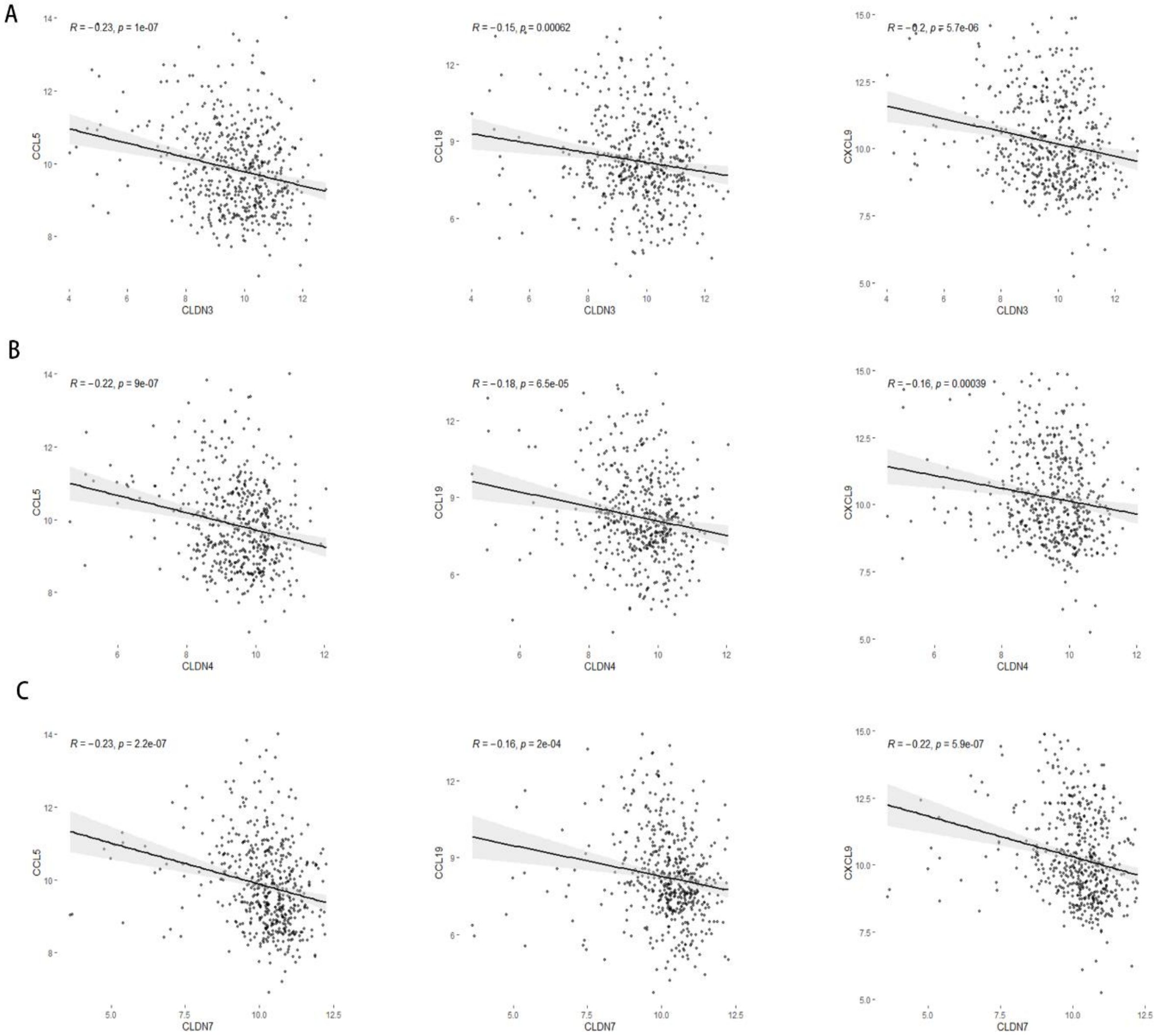

Supplementary Figure 4. Correlations between expression level of cytokines and claudins in GSE25066 dataset. (A) Negative correlations between CLDN3 and CCL5/CCL19/CXCL9 expression. (B) Negative correlations between CLDN4 and CCL5/CCL19/CXCL9 expression. (C) Negative correlations between CLDN7 and CCL5/CCL19/CXCL9 expression.

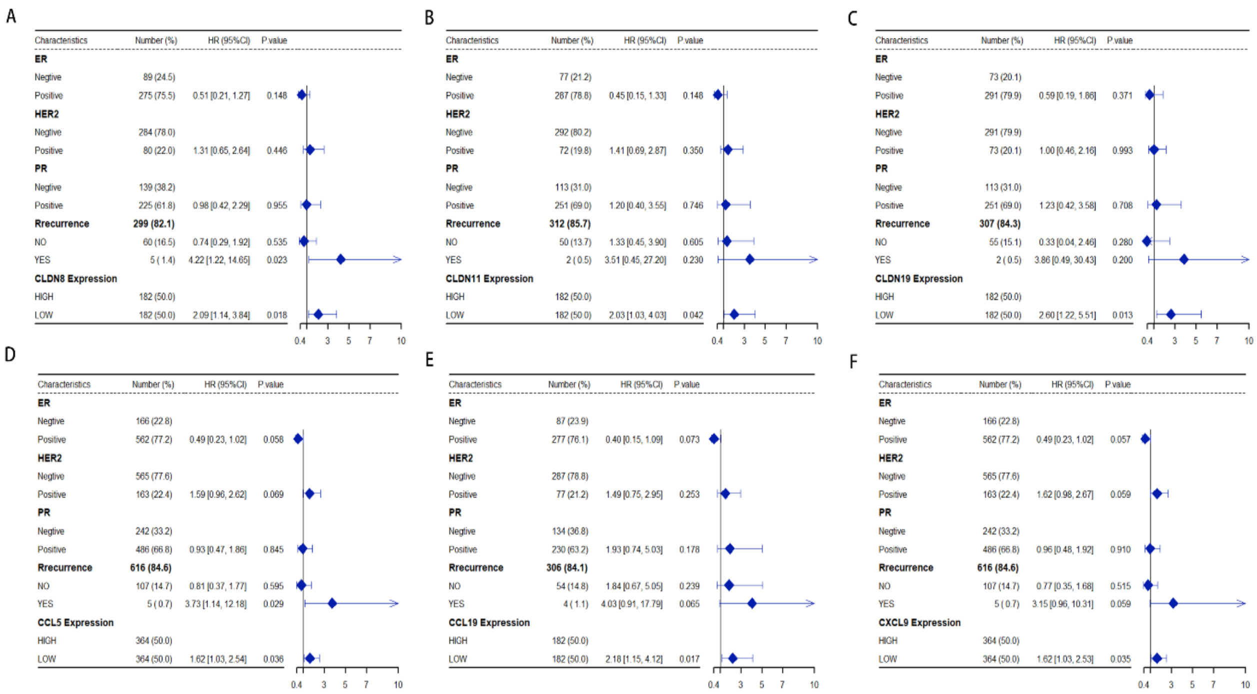

Supplementary Figure 5. Multivariable Cox regression model used for predicting the outcome of BCa patients in TCGA-BRCA. The association of each variable (featured claudins or cytokines) with the outcome of BCa patients by Cox regression analyses: low expression of CLDN8 (A), CLDN11(B) and CLDN19(C) indicated poor outcome of BCa (HR>1.0); low expression of CCL5 (D), CCL19 (E) and CXCL9 (F) indicated poor outcome of BCa (HR>1.0). Abbreviations: HR = Hazard Ratio, CI = Confidence Interval, No. = Number.
